# Virtually Walking in a Patient’s Shoes—the Path to Empathy?

**DOI:** 10.1007/s40670-020-01101-0

**Published:** 2020-10-10

**Authors:** Carrie A. Elzie, Jacqueline Shaia

**Affiliations:** Department of Pathology and Anatomy, Eastern Virginia Medical School, Norfolk, VA USA

**Keywords:** Virtual reality, Empathy

## Abstract

Empathy is the basis of a patient-physician relationship; however, this is being lost by students throughout medical training. Immersive virtual reality that allows individuals to viscerally experience anything from another person’s point of view has the potential to reverse the erosion of empathy and improve clinical practices.

Empathy is arguably the “backbone” of the patient-physician relationship, as it has been shown to have numerous positive clinical outcomes for both the patient and the provider. Empathetic care giving is associated with improved patient satisfaction, compliance, and outcomes; clinical competence, career satisfaction, and burnout reduction; as well as diminished medical errors and litigation claims [1]. Unfortunately, previous studies have shown erosion in empathy and compassion during both medical school and residency training, with more recent studies showing a less bleak forecast [2]. As such, various pedagogical methods including balint groups, small groups, workshops, humanities exposure, standardized patient encounters, roleplaying, and reflection have been employed to improve empathetic skills with varying success [3]. However, there is no current standard for empathy/compassion training within medical education. Given these findings, it is timely and important to look at innovative teaching platforms for enhancing and sustaining empathy in medical and health professional education.

As an experiential learning platform, virtual reality has been dubbed the “ultimate empathy machine” as it has been shown to invoke empathy through a process of total embodiment which allows users to virtually step into the shoes of others and see the world from their perspective. In addition to generating empathy, virtual body transfers have been shown to reduce cognitive biases, adopt new attitudes, provoke behavioral responses, increase self-compassion, and influence career choices [4]. Compared to training with people (peers, standardized patients, or patients), virtual reality software may be more accessible, reliable, and economical in the long run. One such promising commercial software, Embodied Labs (https://embodiedlabs.com), allows users to “become” different patients with terminal lung cancer, Alzheimer’s disease, Parkinson’s disease, or macular degeneration [5]. Using Oculus Rift virtual reality goggles, users embody these patients as they undergo the health care process, experience disease symptoms, navigate family dynamics, and engage with various care teams. Thus far, we have piloted three labs to gather feedback on the usability, feasibility, and efficacy of this tool as an overall learning platform utilizing the company’s standard pre/post survey tools and asking about overall learning experience with an internal questionnaire (Table 1). Overall, students have been greatly satisfied with the experience, rating it as a highly valuable learning experience and requesting incorporation into future curricula. Only a few students with very low visual acuity or with a severe motion sickness were unable to complete the modules using the virtual reality goggles. Results on post surveys generated by the software indicated a self-reported gain in understanding of the disease process, what patients endure, and the challenges experienced by family members. In feedback and focus groups, we noticed a high level of immersion indicated by first-person language (as if they were actually the patient), empathetic discourse about the patient and family members, and a change in emotional perspectives. Our preliminary results warrant further exploration with respect to empathy, utilization as a clinical skills training tool, and long-term benefits.

**Table 1:** VR patient experiences and student evaluation themes

	Clay Lab	Beatriz Lab	Dima Lab
Medical theme	Terminal cancer	Alzheimer’s disease	Parkinson’s disease
Patient experience	Observe a professional delivering a serious diagnosis, hospice care team, end-of-life support for the patient and family, and eventual death. Inclusion of a skills lab to practice delivering serious news	Latina woman’s transition from mild to severe forms of the disease, experience hallucinations, memory loss, confusion, and family conflicts regarding care	Lebanese American of Muslim faith experiences hallucinations, falls, sound sensitivity, as well as family communication. Cultural needs are explored throughout the health care field
Pilot group & data collection	154 first year medical students as part of the general mechanisms of disease course (pre/post surveys)	22 medical and physician assistant students as part of the geriatric interest group (pre/post surveys)	6 contemporary human anatomy graduate students as part of the neuroanatomy course (focus group)
Overall experience	84.2% of students felt like they had a better understanding of what patients with terminal cancer experience after completing the lab. 93% of students thought the experience was valuable and 84% wanted to see it incorporated formally into the curriculum	86% of students felt like they had a better understanding of what patients with Alzheimer’s disease experience after completing the lab. 100% of students thought the experience was valuable and wanted to see it incorporated into the curriculum	100% of students felt like they had a better understanding of what patients with Parkinson disease experience after completing the lab. 100% of students thought the experience was valuable and wanted to see it incorporated into the curriculum
Sample quote	“I felt hurt. I felt disappointed and helpless. Looking into the doctor’s eyes as she delivered the news was a powerful experience. I could feel how the energy of the room changed and the emotions of everyone who was there. I saw the reactions of my wife and my daughter.”	“I was mad, frustrated and afraid at my inability to do things I had been doing my whole life. How could my family seem so angry with me, as if it were my fault!”	“I felt frustrated that I could not do the things I used to be able to do effortlessly, such – i.e. writing, walking to the garden, hanging out in the living room with the (grand)kids.”

With virtual reality software and the development of artificial intelligence, the old adage of walking a mile in someone else’s shoes is now possible and should be a part of requisite medical and health professional training. As stand-alone experiences, they can provide powerful emotional responses related to understanding, empathy, and compassion towards patients and their family members. Moreover, such experiences could be coupled with clinical skills training, such as history taking and communication strategies, to determine the extent to which they can influence clinical practice. Given the portability of the technology, similar experiences could be brought into practice settings for residents, physicians, and other health care professionals. This technology could even be extended into the community and used by leaders, emergency response personnel, health care staff, informal caregivers, and support groups. Furthermore, this can be used as a distance teaching tool through software mirroring that would allows a singular user to share their user experience with a large group of distance learners. Thus, the potential of virtual reality to improve the compassion crisis within health care seems limitless.

## Data Availability

All data generated or analyzed during this study are included in this published article.
